# A Theory-Based Analysis of COVID-19 Vaccine Hesitancy among African Americans in the United States: A Recent Evidence

**DOI:** 10.3390/healthcare9101273

**Published:** 2021-09-27

**Authors:** Manoj Sharma, Kavita Batra, Ravi Batra

**Affiliations:** 1 Department of Environmental and Occupational Health, University of Nevada, Las Vegas, NV 89119, USA; manoj.sharma@unlv.edu; 2 Office of Research, Kirk Kerkorian School of Medicine, University of Nevada, Las Vegas, NV 89102, USA; 3 Department of Information Technology, Coforge Ltd., Atlanta, GA 30338, USA; ravi.batra123@gmail.com

**Keywords:** COVID-19, pandemic, SARS-CoV, African American, multi-theory model, COVID-19 vaccine, vaccine hesitancy

## Abstract

African Americans have been disproportionately vaccinated at lower rates, which warrants the development of theory-based interventions to reduce vaccine hesitancy in this group. The fourth-generation theories, e.g., multi-theory model (MTM) of health behavior change, are vital in developing behavioral interventions. Therefore, the current study aims to determine recent trends in COVID-19 vaccination rates and to test the MTM model in predicting the initiation of COVID-19 vaccines among vaccine-hesitant Blacks. A sample of 428 unvaccinated African Americans were recruited through a web-based survey using a 28-item psychometric valid questionnaire. Chi-square, independent-samples-t-test or Welch’s t test, and Pearson’s correlation tests were utilized for the analyses. Hierarchical regression modelling was performed to determine the increment in variation accounted for through addition of predictors over a set of models. Nearly 48% of unvaccinated Blacks reported being vaccine-hesitant. The vaccine-hesitant group was relatively younger (40.5 years ± 15.8 vs. 46.2 years ± 17.4, *p* < 0.001), were Republicans (22.1% vs. 10.0%, *p* < 0.001), lived in the North-East region (26.0% vs. 11.4%, *p* < 0.001) and had religious affiliations other than Christianity (21.2% vs. 13.6%, *p* = 0.04). The mean scores of perceived advantages ((9.01 ± 3.10 vs. 7.07 ± 3.60, *p* < 0.001) and behavioral confidence (8.84 ± 3.76 vs. 5.67 ± 4.09, *p* < 0.001) were higher among vaccine non-hesitant group as opposed to the hesitant ones. In a final regression model, all MTM constructs) predicted nearly 65% of variance in initiating COVID-19 vaccination behavior among the vaccine-hesitant group (adjusted R^2^ = 0.649, F = 32.944, *p* < 0.001). With each unit increment in MTM constructs (e.g., participatory dialogue and behavior confidence), the initiation of COVID-19 vaccination among vaccine-hesitant Blacks increased by 0.106 and 0.166 units, respectively. Based on the findings of this study a m-health educational intervention to promote COVID-19 vaccine uptake behavior among Blacks is proposed.

## 1. Introduction

The COVID-19 pandemic is an ongoing public health threat, which accelerated the development of the COVID-19 vaccine to prevent severe illness, hospitalizations, and mortality [1]. In May 2020, a partnership between the Departments of Health and Human Services and Defense established the Operation Warp Speed (OWS) with a projected goal of producing 300 million doses by early 2021 [2]. In the United States, on 11 December 2020, Pfizer’s BioNTech (BNT161b2) vaccine was approved by the Food and Drug Administration (FDA) as the first vaccine for emergency use for people 16 years and older [3]. Then, a week later, Moderna’s (mRNA-1273) vaccine was approved by the FDA followed by the approval of single-shot Johnson and Johnson’s vaccine (Ad26.COV2. S) on 27 February 2021 for people 18 years and older [3]. In clinical studies, all three vaccines have shown sufficient efficacy in preventing COVID-19 infection [4,5,6]. Given the vaccine was approved on an emergency use authorization (EUA) basis on a fast approval track, there are some concerns and hesitancy among the general public regarding the COVID-19 vaccine uptake behavior. Attitudes of Antivaxxers, myths and misconceptions, conspiracy theories, the unknown nature of long-term side effects, and prevailing skepticism have also been fueling the vaccine hesitancy [7,8,9,10]. As of 25 August 2021, nearly 203 million or 60.1% of the population has been fully or partially vaccinated in the U.S. [11]. This is short of the Biden administration’s projected goal of giving at least one COVID-19 vaccine shot to 70% of the U.S. adult population by 4 July 2021 [12].

Recent studies indicated that COVID-19 vaccine hesitancy is higher among Blacks or African Americans [13]. A pooled analysis of 13 studies found COVID-19 vaccine hesitancy to be 26.3% in all Americans and 41.6% (95%CI: 34.4–48.9) for Blacks [14]. Previous studies have voiced concerns about racial differences in mistrust as a contributing factor of hesitancy among Blacks towards vaccines as well as clinical research [15]. In addition, reduced access to healthcare, less research evidence with African American participants in studies, lower awareness and educational attainment, and history of the Tuskegee Syphilis study’s ethical misconduct were also cited as contributing factors of vaccine hesitancy [16,17,18,19]. This is alarming as Blacks share a disproportionate burden of high morbidity, mortality, and socioeconomic impacts of COVID-19 pandemic [20]. These disparate effects were also existent during the 2009 influenza outbreak and vaccination; the current event (COVID-19 pandemic) is a wake-up call to address this discrepancy and gain trust of communities hit hard by the pandemic [21]. Until today, no theory-based studies have been conducted to understand the correlates of COVID-19 vaccine hesitancy among Blacks or African Americans, which will aid in developing evidence-based interventions to promote vaccine acceptability in this group.

A theoretical basis is vital in deciphering why people engage or fail to engage in behaviors that promote health [22]. The theoretical evidence can help designing efficacious, effective and precision interventions [23]. Theory-based health behavior research has evolved over the years from crude knowledge–attitude–practices (KAP) surveys, to skill-based intervention planning, to single theory interventions to the current Fourth-generation multiple theory precision interventions [23]. One such Fourth-generation theory is the multi-theory model (MTM) of health behavior change [23,24], which will be the framework of this proposed study. Therefore, the purpose of this study was to identify sociodemographic and MTM-based differences between vaccine-hesitant African Americans and non-vaccine-hesitant African Americans. Further, the study aimed to utilize MTM in explaining COVID-19 vaccine acceptability behavior among Blacks from data collected during the third quarter of 2021 and proposed an m-health (mobile phone-based) educational intervention for the possible implementation.

### 1.1. Study Design and Participants

This study was a cross-sectional survey including 28 questions. The survey was conducted during July-August 2021 to recruit unvaccinated African American adults residing in the United States. According to the recent estimates, the African American racial subgroup constitutes 13.5% of the U.S. population [25]. Reportedly, African American subgroups were less willing to take the COVID-19 vaccine than their other race counterparts [26,27].

### 1.2. Recruitement and Data Collection

A commercial survey sampling and administration company, Qualtrics, was contacted to recruit a nationwide sample of unvaccinated African American U.S. residents [28,29]. The majority of the sample come from the traditional market research panels, however, for hard-to-reach or restrictive sample (such as that used in this study), Qualtrics uses a niche panel through specialized campaigns. Invitation methods include but are not limited to emails and in-app notifications. Panel members can unsubscribe from the emails at any time [28,29]. Screening questions are asked from the potential participants prior to survey entry to ensure eligibility [28,29]. To prevent the self-selection bias, survey invitation was kept general and specific details of the survey were not revealed before the screening questions. The respondents were compensated for their time to take the survey and incentives may include gift cards, redeemable points, cash rewards, vouchers, SkyMiles, etc.

### 1.3. Ethical Considerations

This study was conducted according to the Helsinki declarations. The research protocol and informed consent forms used in this study were approved by the Institutional Review Board (IRB) of the University of Nevada, Las Vegas (IRB Protocol # 1702350-2). A detailed information sheet outlining the objectives, procedures of the study, duration of the survey and expected outcomes including benefits, risks, and dissemination of findings were provided to the participants before taking the survey. The participation in this survey was made completely voluntary by providing options of “agree” or “disagree” to the participants. Adherence to the Health Insurance Portability and Accountability Act (HIPAA) was maintained during this study. In other words, no personal identifiers were collected to preserve anonymity of the responses. Each participant was allowed to take the survey only once to prevent ballot box stuffing, which was the algorithm used during the data collection. To exclude duplication and ensure validity, Qualtrics uses the unique digital fingerprinting mechanism to retain the integrity of the survey data.

### 1.4. Survey Instrument

We used a psychometric valid and reliable tool developed/used by a previous study to explain COVID-19 vaccine hesitancy among U.S. college students [30]. This tool was based on the Fourth-generation theory called the Multi-theory Model (MTM) framework [23,24]. The MTM has been widely tested in studying or explaining several health behaviors such as physical activity [31], and fruits and vegetables intake [32]. In addition, the MTM has also been used to explain behaviors (e.g., handwashing) and social connectedness during the COVID-19 pandemic [33,34]. The survey tool consisted of 15 demographic questions with 13 questions based on MTM subscales of intention to initiate COVID-19 vaccination behavior. A detailed description of the instrument’s components is provided in Table 1. The two MTM constructs of initiation (e.g., Advantages and Disadvantages) were measured on a 5-point Likert scale, which ranges from “Never” to “Very Often.” As described in the Table 1, the Participatory dialogue score was obtained after subtracting the total disadvantages score from the advantages score of each participant. The other two constructs, i.e., behavioral confidence and changes in the physical environment were measured on a scale of surety, which ranged from “Not at all sure” to “Completely sure” on a 5-point Likert scale (Table 1). Each construct has 3 items and the score ranges from 0–12 units.

### 1.5. Statistical Analysis

**Figure 1:**
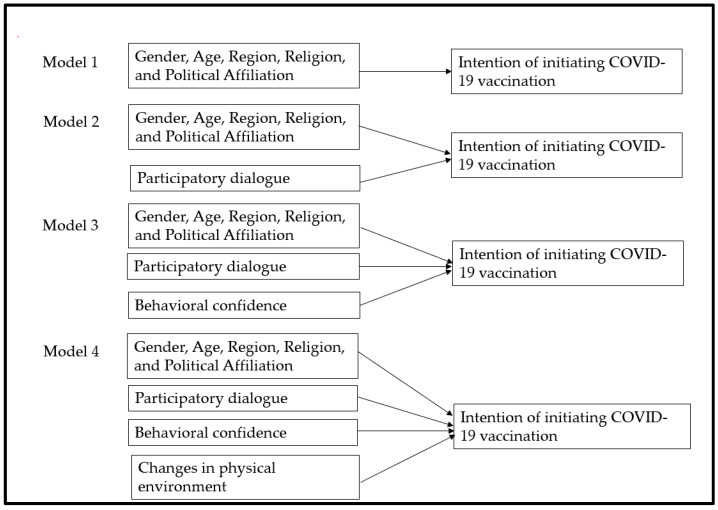
Regression model building process indicating hierarchical entry of predictors to explain variance in the dependent variable (intention of initiating COVID-19 vaccination).

All analyses were conducted using IBM SPSS v.26, (IBM Corp., Armonk, NY, USA) and SAS 9.3 (SAS Institute, Cary, NC, USA). Descriptive and exploratory analyses were utilized to investigate data distribution, normality, missing values, and outliers. All assumptions of the statistical tests were assessed. Categorical variables were compared among vaccine-hesitant and non-hesitant groups by Chi-square analyses. The follow-up contingency table analysis (post-hoc) was conducted to obtain *p*-values corresponding to multilevel variables. The observed *p*-values were Bonferroni-corrected in multiple comparisons to prevent type 1 errors [35]. The values of adjusted residuals (or Z scores) were used to generate Bonferroni-corrected *p* values. Effect sizes were reported wherever appropriate. Continuous variables, such as age, advantages, disadvantages, participatory dialogue, behavior confidence, and changes in the physical environment were compared among groups using independent-samples t-tests or Welch’s t test where equal variance could not be assumed. A square root transformation was applied to the non-normally distributed variables, which were later back-transformed for the ease of interpretation. A bivariate Pearson’s correlation was also conducted to investigate the relationships between the MTM constructs. We also calculated the proportion of perceived advantages and disadvantages among hesitant and non-hesitant group. Hierarchical regression modelling was performed to determine the increment in variation (by R-square change) accounted for through addition of predictors over a set of models. The details about the model building process is provided in the Figure 1 given below. The statistical significance was set at 5% for all analyses. For polytomous variables (e.g., region, religion, and political affiliation in our dataset), we used dummy coding by converting the polytomous categorical variable into a series of dichotomous variables for each level where a value of 1 was assigned for each observation at that level and zero for all others. The level of categorical variable which was coded zero at all levels was considered a reference category. The algorithm of the dummy coding is described in the Appendix A Table A1. Prior to running analyses, we conducted power analyses to determine whether our sample was sufficient to detect the hypothesized effects. The following formula was used to compute sample size: *n* = (z)^2^ *p* (1 − *p*)/d2 with a 95% confidence interval (alpha = 0.05, z = 1.96), and a margin of error d = 5%, and the proportion of vaccine hesitancy among Blacks was 18% in May 2021 based on the data reported by the Office of the Assistant Secretary for Planning and Evaluation Report [36]. The estimated sample size was 227 and the true sample size was increased to 428 allow group comparisons. We used a Checklist for Statistical Assessment of Medical Papers (CHAMP statement) for our results’ reporting [37].

## 2. Results

**Figure 2:**
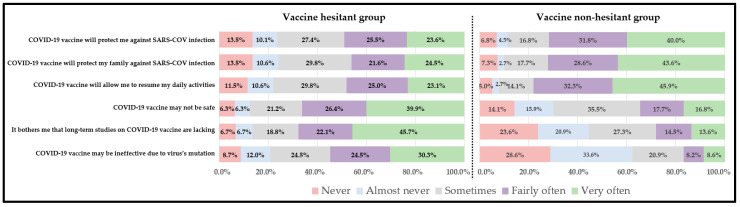
Perceived advantages and disadvantages of COVID-19 vaccination among hesitant and non-hesitant group.

Nearly 48 percent of our sample population (208 out of 428) reported that they are hesitant to take the COVID-19 vaccine (Table 2). The vaccine-hesitant group was relatively younger compared to the vaccine non-hesitant group (40.5 years ± 15.8 vs. 46.2 years ± 17.4, *p* < 0.001, Table 2). There was no statistically significant differences in the proportion by gender, education, marital status, employment status and location of residence. However, the vaccine-hesitant group had a significantly higher proportion of Republicans (22.1% vs. 10.0%, *p* > 0.001), those living in the North-East region (26.0% vs. 11.4%, *p* < 0.001) and belong to religions other than Christianity (21.2% vs. 13.6%, *p* = 0.04, Table 2). As expected, there were significant differences in the proportions of perceived advantages and disadvantages among vaccine-hesitant and vaccine non-hesitant group (Figure 2). Over 40% of the vaccine non-hesitant group strongly perceived the advantage of the COVID-19 vaccine in protecting them or their family members against SARS-CoV infection compared to nearly 20.0% among the vaccine-hesitant group (Figure 2). Nearly 40% of the vaccine-hesitant group perceived COVID-19 to be unsafe compared to only 16.8% among the vaccine non-hesitant group. The vaccine-hesitant group was more concerned about the lack of long-term studies on the vaccine’s effectiveness (45.7% vs. 13.6%) and the virus’s mutation, which may lead to vaccine breakthrough (30.3% vs. 8.6%, Figure 2). The mean score of perceived advantages was significantly higher among the vaccine non-hesitant group compared to the vaccine hesitant group (9.01 ± 3.10 vs. 7.07 ± 3.60, *p* < 0.001, Table 3). On the contrary, the mean score of perceived disadvantages was higher among the vaccine-hesitant group compared to the non-hesitant group (8.36 ± 3.02 vs. 5.15 ± 3.12, *p* < 0.001, Table 3). The vaccine non-hesitant group had a statistically significant higher mean score of the behavior confidence as compared to the hesitant group (8.84 ± 3.76 vs. 5.67 ± 4.09, *p* < 0.001, Table 3). The results of Pearson’s correlation test indicated a moderate significant positive correlation between the constructs of advantages, behavior confidence and changes in the physical environment (*p* < 0.001, Table 4). A strong positive correlation was also found between the physical environment and behavior confidence (*p* < 0.001). In hierarchical regression analysis, the overall regression model (with age and all MTM constructs) predicted nearly 65% of variance in initiating COVID-19 vaccination behavior among the vaccine-hesitant group (Adjusted R^2^ = 0.649, F = 32.944, *p* < 0.001, Table 5). With each unit increment in participatory dialogue and behavior confidence, the conditional mean for initiation of COVID-19 vaccination among hesitant Blacks increased by 0.106 and 0.166 units, respectively (Model 4, Table 4). Among the demographic variables, except age, the slope for gender, region, religion, and political affiliation were not significant, indicating no significant differences in the conditional mean change in the initiation among vaccine-hesitant Blacks.

## 3. Discussion

The purpose of this study was to identify correlates of vaccine hesitancy among African Americans, including those derived from MTM, and upon successful testing of MTM present a framework for the m-health intervention. In our study, 48.6% African Americans had not been vaccinated and expressed vaccine hesitancy. The study found that, among socio-demographic characteristics, age (younger Blacks being more hesitant), being from the Northeast region of the United States, being a Republican as political affiliation, and having a religion other than Christianity or atheist had statistically significant greater proportion of vaccine-hesitant Blacks. Previous reports have indicated that Blacks bear a disproportionate burden of COVID-19-associated morbidity and mortality, which in turn contribute to vaccine hesitancy among this group [38]. Blacks have distrust towards medical profession and research, which might explain hesitancy of this group [38]. While testing MTM constructs, it was noted that all constructs of MTM were statistically significant between vaccine-hesitant Blacks and vaccine non-hesitant Blacks with effect sizes ranging predominantly from medium to large with the exception of participatory dialogue where the effect size was small. The MTM constructs of participatory dialogue and behavioral confidence along with age accounted for a large proportion of variance (64.9%) in explaining the likelihood of getting the COVID-19 vaccination among the hesitant African Americans. Thus, this established MTM as a robust model for m-health intervention planning.

Upon examining our results in the context of existing literature, age was an important determinant of vaccine hesitancy with younger Blacks being more hesitant than their older counterparts. This finding is also supported by the study by Fisher and colleagues (2020), who early on in the pandemic found that younger age was associated with greater vaccine hesitancy [39]. Similar evidence for greater vaccine hesitancy among the younger population was also found by other studies [40,41,42]. This can be explained by the preconceived notion of the younger generations of being ‘invincible’, beliefs of toughness, not enough confidence in vaccines, mistrust with the medical system especially among African Americans [43], carelessness, or a carefree attitude. These findings suggest developing concerted efforts for COVID-19 vaccine promotion among Black youth through diverse channels.

Next, vaccine-hesitant African Americans were clustered in the Northeastern region of the U.S. This was somewhat surprising as the Southern region was presumed to have a greater proportion of vaccine-hesitant groups given the lowest rates of vaccination per previous studies [44]. The reason for this could be that while the Northeastern region has the highest rates of vaccination, this has not still reached total vaccination coverage and the remaining group of people may be laggards, thus driving up the percentage of COVID-19 vaccine hesitancy in this subgroup of African Americans. Further, the Pew Research Center (2021) reported that the Black population in the U.S. is growing and is becoming more diverse [45]. From 2000 to 2019, there was a 29% increase in the African American population [45]. Moreover, nearly 10% of African Americans were foreign-born, with New York (area that lies in Northeast U.S.) having the largest number [45]. A substantial proportion of this Black population that is foreign-born mainly from the Caribbean [45]. Some of the cities in the Northeast have a greater number of such individuals and they may possibly be more reluctant to get vaccinated as they may not have assimilated into the American culture [45]. These findings about greater disparity in the North-East has implications for targeting African Americans residing in the North-East region with proactive educational campaigns and not becoming complacent.

Consistent with previous reports, the Republican political affiliation had greater proportion of vaccine hesitancy as opposed to those being Democrats [13,46,47,48,49]. These findings suggest the need for Republican party leaders and especially African American Republican role models to support openly the COVID-19 vaccine campaigns.

Religion in the African American community other than Christianity and Atheist was found to have higher vaccine hesitancy (21.2%) in comparison to the non-hesitant group (13.6%). The predominant other religion within the African American community is Islam [49]. A majority of these Muslim African Americans live in the Northeast [50]. Our study found a greater proportion of African Americans with vaccine hesitancy living in the Northeast and this could be an attribute related to that distribution.

As expected, our study found all MTM constructs to be statistically significant and having higher scores for the non-hesitant African American population as compared to the hesitant population. Furthermore, the constructs of participatory dialogue and behavioral confidence were statistically significant predictors of the likelihood of taking the COVID-19 vaccine among the hesitant group. No previous study using MTM has been conducted in this regard with the African American community and this was the first study. However, a previous study has been done with college students in which all three constructs of MTM were found to be significant predictors and accounted for 54.8% of variance in the likelihood of taking the COVID-19 vaccine among vaccine-hesitant students [30]. In our study, we did not find changes in the physical environment as significant because the previous study was done when the vaccine was not available, but at present availability is no longer an issue and the COVID-19 vaccine is available to anyone intending to take it. In our sample, 96.6% (201/208) of the vaccine-hesitant group had a cell phone. Based on the results of this study, an m-health (mobile phone based) educational intervention for testing and implementation with Blacks to promote COVID-19 vaccine uptake behavior can be undertaken. Therefore, based on these findings an m-health intervention can be proposed.

### 3.1. Proposition of a m-Health (Mobile Phone-Based Intervention)

**Figure 3:**
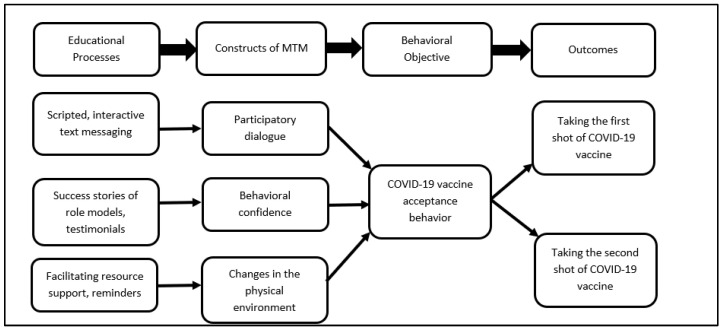
Diagrammatic depiction of how the multi-theory model (MTM) for health behavior change can be used to improve the COVID-19 vaccine uptake behavior through an m-health intervention.

Based on the findings of this survey, m-health (mobile phone-based) interventions can be planned and instituted. This can be a brief and precise intervention. The tentative logic model for the intervention is depicted in Figure 3. For designing and implementing such an intervention, a variety of educational methods would need to be utilized such as scripted, interactive text messaging that would underscore the advantages of the COVID-19 vaccine over the disadvantages and initiate participatory dialogue between the facilitator and the client. This will help the participants sway their decisions towards greater vaccine acceptance. In order to build behavioral confidence, success stories of role models and testimonials would need to be shared with the clients. For instance, messages from leading Black health professionals and community leaders can encourage COVID-19 vaccine uptake behavior among the African American community. For changes in the physical environment, even though the vaccine is available, reminder systems and facilitation of resources would need to be undertaken to ensure that the immunization schedule is completed, including the proposed boosters. It is critical to help the participants overcome logistical barriers in getting the vaccine that can be provided through phone dialogue linking people to resources.

### 3.2. Strengths and Limitations

To our knowledge, this study is the first study to utilize a contemporary theoretical paradigm of MTM to explain the correlates of COVID-19 acceptability among a nationally representative sample of African Americans. The study also delved into understanding the differences between COVID-19 vaccine-hesitant African Americans and non-vaccine-hesitant African Americans. The study found evidence in support of MTM and was able to propose a putative m-health intervention for possible implementation in high need areas. However, the study also had some limitations. First, the cross-sectional study design limits the causal inferencing due to measurement of independent and dependent variables at the same point in time. Second, we might expect some residual confounding attributed to unmeasured variables, for example academic performance. Third, self-reports are the only means of gathering information about attitudes, and they suffer from measurement bias in the form of dishonesty, exaggeration, yay saying, nay saying, etc. Moreover, the likelihood of getting the COVID-19 vaccine was measured through self-report as a proxy of actual behavior. Therefore, these limitations need to be considered while interpreting the results for causality.

## 4. Conclusions

Our study found that a substantial proportion of African Americans who have not yet been vaccinated have a high degree of vaccine hesitancy (48.6%). Our study also found that age (younger Blacks being more hesitant), residence in North-East region of the United States, having a Republican political affiliation, and belonging to a religion other than Christianity or atheist resulted in statistically significant greater proportion of vaccine-hesitant African Americans. Our study found that all the constructs of MTM were statistically significant between vaccine-hesitant African Americans and non-vaccine-hesitant African Americans. The MTM constructs of participatory dialogue and behavioral confidence along with age explained a large proportion of variance in the likelihood of getting COVID-19 vaccination among the hesitant African Americans. The study proposed a framework for an m-health intervention for African Americans.

## Figures and Tables

**Table 1:** Theoretical framework of Multi-level Theory Model explaining the initiation of COVID-19 vaccination.

Domain	Constructs		Description (s)
**Intention of initiating COVID-19 vaccination**	Participatory dialogue (Difference of advantages and disadvantages)	Advantages	Perceived advantages of COVID-19 vaccination
		Disadvantages	Perceived disadvantages of COVID-19 vaccination
	Behavioral confidence		Confidence of taking COVID-19 vaccination despite external and internal restrictive factors
	Changes in the physical environment		COVID-19 vaccination by facilitating enabling factors from the environment

**Table 2:** Demographic characteristics of the sample population (*n* = 428).

Variable Name	Categories	Overall Sample	COVID-19 Vaccine Hesitancy		Test Statistics	*p* Value	Effect Size
			**Yes (*n* = 208)**	**No (*n* = 220)**			
**Age (Mean ± SD)**	-	43.43 ± 16.9	40.5 ± 15.8	46.2 ± 17.4	−3.475 *	**0.001**	0.34
**Gender**	Male	203 (47.4)	101 (48.6)	102 (46.4)	1.202	0.5	0.053
	Female	225 (52.6)	107 (51.4)	118 (53.6)			
**Marital status**	Divorced/Separated/Widowed	70 (16.4)	30 (14.4)	40 (18.2)	6.822	0.07	0.126
	Married	195 (45.6)	106 (24.8)	89 (20.8)			
	Never married	120 (28.0)	41 (23.6)	79 (32.3)			
	Other	43 (10.0)	23 (11.1)	20 (9.1)			
**Education**	High school diploma or GED	68 (15.9)	30 (14.4)	38 (17.3)	5.048	0.7	0.109
	Associate degree	46 (10.7)	20 (9.6)	26 (11.8)			
	Bachelor degree	96 (22.4)	45 (21.6)	51 (23.2)			
	Master degree	68 (15.9)	37 (17.8)	31 (14.1)			
	Doctoral degree	18 (4.2)	9 (4.3)	9 (4.1)			
	Professional degree	14 (3.3)	5 (2.4)	9 (4.1)			
	Some college but no degree	88 (20.6)	47 (22.6)	41 (18.6)			
	Trade school	15 (3.5)	6 (2.9)	9 (4.1)			
	Less than a high school diploma	15 (3.5)	9 (4.3)	6 (2.7)			
**Annual Income**	Less than $25,000	87 (20.3)	38 (18.3)	49 (22.3)	3.910	0.7	0.096
	$25,001–$50,000	89 (20.8)	42 (20.2)	47 (21.4)			
	$50,001–$75,000	76 (17.8)	38 (18.3)	38 (17.3)			
	$75,001–$100,000	45 (10.5)	25 (12.0)	20 (9.1)			
	$100,001–$125,000	42 (9.8)	22 (10.6)	20 (9.1)			
	$125,001–$150,000	39 (9.1)	22 (10.6)	17 (7.7)			
	$150,000+	50 (11.7)	21 (10.1)	29 (13.2)			
**Employed**	Yes	273 (63.8)	139 (66.8)	134 (60.9)	1.621	0.2	0.062
	No	155 (36.2)	69 (16.1)	86 (20.1)			
**Region**	Midwest	94 (22.0)	44 (21.2)	50 (22.7)	16.322	0.7	0.195
	Northeast	79 (18.5)	54 (26.0)	25 (11.4)		**<0.001**	
	South	164 (38.3)	72 (34.6)	92 (41.8)		0.1	
	West	90 (21.0)	38 (18.3)	52 (23.6)		0.2	
**Political affiliation**	Democrat	276 (64.5)	116 (55.8)	160 (72.7)	15.923	**<0.001**	0.193
	Republican	68 (15.9)	46 (22.1)	22 (10.0)		**<0.001**	
	Others, including independent	84 (19.6)	46 (22.1)	38 (17.3)		0.2	
**Religion**	Christianity	309 (72.2)	147 (70.7)	162 (73.6)	5.734	0.5	0.116
	Atheist	45 (10.5)	17 (8.2)	28 (12.7)		0.13	
	Others	74 (17.3)	44 (21.2)	30 (13.6)		**0.04**	
**Location of residence**	Urban	240 (56.1)	114 (54.8)	126 (57.3)	2.60	0.3	0.078
	Semi urban	122 (28.5)	56 (26.9)	66 (30.0)			
	Rural	66 (15.4)	38 (18.3)	28 (12.7)			

Note: The percentages may not add up to 100% as some of the participants preferred not to answer a few questions. *p* values for multiple comparisons are Bonferroni-corrected unless overall *p* values came insignificant; * T statistics.

**Table 3:** Comparison of Multi-theory model (MTM) constructs among vaccine-hesitant and non-hesitant groups.

MTM Construct	Vaccine Hesitancy		*p* Value	Effect Size (Cohen d)
	Yes (*n* = 208)	No (*n* = 220)		
Overall Initiation Score	1.68 ± 1.47	3.06 ± 1.30	<0.001	0.9 [Large]
Subscales				
Perceived Advantages	7.07 ± 3.60	9.01 ± 3.10	<0.001	0.6 [Medium]
Perceived Disadvantages	8.36 ± 3.02	5.15 ± 3.12	<0.001	1.0 [Large]
Participatory Dialogue	1.66 ± 1.08	4.12 ± 1.01	<0.001	0.2 [Small]
Behavior Confidence	5.67 ± 4.09	8.84 ± 3.76	<0.001	0.8 [Large]
Changes in the Physical Environment	7.36 ± 3.65	9.21 ± 3.50	<0.001	0.5 [Medium]

Note: All measures are represented as mean ± standard deviation unless stated otherwise. Square root transformation was applied to non-normally distributed variables (e.g., Participatory Dialogue). Another reason to transform the PD was to convert negative values to positive for the ease of interpretation. The transformed variable was then back-transformed for easy interpretation. The calculated effect size was categorized as small, medium, and large according to the Cohen’s convention.

**Table 4:** Pearson correlations, and reliability estimates for study variables in the sample population (*n* = 428).

Variables	1	2	3	4	5
1. Advantages	-	0.27 **	0.64 **	0.57 **	0.15 **
2. Disadvantages	0.27 **	-	00.31 **	−0.18 **	0.15 **
3. Behavioral Confidence	0.64 **	0.31 **	-	0.76 **	0.21 **
4. Physical Environment	0.57 **	0.18 **	0.76 **	-	0.24 **
5. Age	0.15 **	0.15 **	0.21 **	0.24 **	-
Mean	8.07	6.71	7.3	8.3	43.43
Standard Deviation	3.5	3.4	4.2	3.7	16.9
α	0.91	0.77	0.91	0.93	-

** *p* < 0.01; The Cronbach alpha value of the entire scale is 0.82.

**Table 5:** Hierarchical Multiple Regression (HRM) predicting likelihood for initiation of COVID-19 vaccination among hesitant African Americans (*n* = 208).

Variables	Model 1		Model 2		Model 3		Model 4	
	B	*β*	B	*β*	B	*β*	B	*β*
**Vaccine-hesitant group (*n* = 228)**								
Constant	1.748		2.452		1.538		1.355	
Age	−0.020 *	−0.214	−0.019 **	−0.199	−0.018 **	−0.195	−0.018 **	−0.193
**Gender (ref: Female)**								
Male	0.332	0.112	−0.057	−0.019	−0.017	−0.006	−0.029	−0.010
**Political Affiliation (ref: Republicans)**								
Democrats	0.739	0.249	0.317	0.107	0.074	0.025	0.069	0.023
Other, including independent	−0.128	−0.036	−0.331	−0.093	−0.194	−0.055	−0.222	−0.062
**Religion Affiliation (ref: Christianity)**								
Atheist	−0.288	−0.054	−0.157	−0.029	0.262	0.049	.238	0.044
Others	0.399	0.110	0.343	0.095	0.159	0.044	0.164	0.045
**Region (ref: South)**								
Midwest	0.349	0.097	0.235	0.065	−0.036	−0.010	0.002	0.010
West	0.165	0.043	0.073	0.019	−0.284	−0.074	−0.245	−0.064
Northeast	0.155	0.046	0.036	0.011	−0.196	−0.058	−0.144	−0.043
**MTM Constructs**								
Participatory dialogue	-	-	0.169 **	0.587	0.106 **	0.367	0.106 **	0.370
Behavioral confidence	-	-	-	-	0.192 **	0.531	0.166 **	0.460
Changes in the physical environment	-	-	-	-	-	-	0.042	0.104
R^2^	0.159	-	0.470	-	0.663	-	0.670	-
F	4.156 **	-	17.464 **	-	35.133 **	-	32.944 **	-
Δ R^2^	0.159	-	0.311	-	0.194	-	0.006	-
Δ F	4.156 **	-	115.586 **	-	112.750 **	-	3.648 **	-

* *p*-value < 0.05; ** *p*-value < 0.001; Adjusted R^2^ of initiation in the final model = 0.649.

## Data Availability

The data presented in this study are available on request from the corresponding author. The data are not publicly available due to ethical reasons.

## Appendix A

**Table A1:** Dummy coding algorithm of polytomous variables used in the regression.

Variable	New Variable 1 (X1)	New Variable 2 (X2)	New Variable 3 (X3)
Region			
1 Midwest	1	0	0
2 West	0	1	0
3 Northeast	0	0	1
4 South	0	0	0
Religion			
1 Christianity	0	0	-
2 Atheist	0	1	-
3 Others	1	0	-
Political affiliation			
1 Democrat	1	0	-
2 Republicans	0	0	-
3 Others	0	1	-
